# Immune-molecular nexus in reproductive disorders: mechanisms linking POI and RSA

**DOI:** 10.3389/fgene.2025.1652519

**Published:** 2025-09-29

**Authors:** Chen Chen, Xinyue Zhang, Wenxin Li, Yueqin Liu, Dan Zhao, Subo Zhang, Xiaolan Zhu

**Affiliations:** ^1^ Department of Reproductive Medical Center, Fourth Affiliated Hospital of Jiangsu University (Zhenjiang Maternity and Child Healthcare Hospital), Zhenjiang, Jiangsu, China; ^2^ Department of Radiology, the Second People’s Hospital of Lianyungang City, Lianyungang, Jiangsu, China

**Keywords:** premature ovarian insufficiency, recurrent spontaneous abortion, integrated transcriptomic analysis, machine learning, drug target enrichment

## Abstract

**Background:**

Infertility remains a prevalent global health concern, with Premature Ovarian Insufficiency (POI) and Recurrent Spontaneous Abortion (RSA) being common causes of female infertility.

**Objective:**

This study aims to identify new central genes and potential therapeutic drugs for RSA and POI by integrating multi transcriptome data and machine learning algorithms.

**Methods:**

This study utilized RNA sequencing data from patients with POI and RSA to identify key hub genes associated with these diseases. The analysis involved machine learning algorithms, mcode and Cytoscape, revealing important hub genes. The comprehensive evaluation includes functional annotation, protein-protein interaction (PPI) network, transcription factor (TF) gene regulatory network, microRNA (miRNA) gene regulatory network. Genome enrichment analysis (GSEA) and immune infiltration studies elucidated the potential mechanism between POI and RSA. Drug target enrichment analysis highlighted promising therapeutic agents against RSA and POI. Validation of granulosa cells and endometrial tissue samples using quantitative real-time polymerase chain reaction (qRT-PCR) highlighted the importance of the identified hub genes.

**Results:**

This study identified a total of six hub genes—— CENPW, ENTPD3, FOXM1, GNAQ, LYPLA1, and PLA2G4A. Immunoassay revealed an increase in activated NK cells. Furthermore, significant differences were observed in the proportions of other immune cell types, such as resting memory CD4 T cells, compared to the control group. Significantly, these six genes participate in diverse metabolic pathways linked to RSA and POI, particularly in oxidative phosphorylation, ribosome processes, and steroid biosynthesis pathways. Additionally, ten potential drugs (Rifabutin, Methaneseleninic Acid, Carbamazepine, Dasatinib,Troglitazone, Tamoxifen, Enterolactone, Anisomycin, Testosterone, 5-Fluorouracil) targeting key genes were identifed.

**Conclusion:**

Targeting these genes shows promise for preventing and treating both POI and RSA, providing crucial insights into addressing these complex conditions at molecular level.

## 1 Introduction

As a highly prevalent condition affecting populations globally, infertility has been formally acknowledged by the World Health Organization as a critical public health issue requiring international attention (Boivin et al., 2007). Some studies have noted that the success rate of assisted reproductive technology decreases with increasing age, resulting in a number of embryos facing Recurrent Spontaneous Abortion (RSA) (Wright et al., 2005). Women experiencing RSA may suffer from both emotional distress and physical complications, including diminished endometrial receptivity (Voss et al., 2020; Achache and Revel, 2006). Despite advances in medical research, around 50% of RSA cases are idiopathic, and there is a significant gap in the development of reliable early warning systems and treatment options (Abdollahi et al., 2015). Premature ovarian insufficiency (POI), alongside RSA, is a common factor in female infertility. It triggers short-term complications such as menopausal symptoms and exerts long-term effects on skeletal health, cardiovascular systems, and cognitive performance (Mishra et al., 2021; Webber et al., 2016). It seriously affects women of childbearing age. However, the pathogenesis of POI is complicated and remains unclear. POI and RSA are common, but the etiology and treatment mechanism are not clear. Thus, it is highly important to clarify the pathogenesis of these two diseases and develop preciseiy targeted therapies.

From a hormonal perspective, women with RSA exhibit an increased predisposition to developing POI, as indicated by reduced levels of anti-Müllerian hormone (AMH) and lower Antral Follicle Count (AFC). This association is particularly pronounced in cases of unexplained RSA, where low AMH levels serve as a key indicator (Bunnewell et al., 2020). Scientific findings reveal a distinct immunological link between these two disorders, particularly through the involvement of CD16^−^ and CD56^+^ cells, essential components of the NK cell population. Comparative analyses show decreased levels of these cellular subtypes in both POI and RSA patients relative to healthy controls, indicating common immunological disturbances. These observations are consistent with the autoimmune pathogenesis underlying both conditions (Zhang et al., 2023; Lachapelle et al., 1996). As the most abundant immune cells in the ovaries, macrophages secrete TNF-α, a cytokine highly expressed in POI. This mechanism is implicated in RSA through the regulation of stathmin-1 expression. (Zheng et al., 2023; Guenther et al., 2012). Statistical analysis revealed that the Robertsonian translocation of chromosomes was associated with an increased incidence of POI and RSA (Park et al., 2022; Jiao et al., 2012; Ishizuka et al., 2019). In addition, oocyte-specific genes can control oocyte quality, embryogenesis, and the uterine microenvironment, thereby influencing oocyte superiority and RSA development, and most of these genes are also associated with POI (Dean et al., 2018). Molecular studies have demonstrated that FOXL1 gene mutations in the coding region, identified in POI patients, cause critical substitutions that lead to gene silencing and subsequent alterations in conserved amino acid residues (Harris et al., 2002). The FOXL2 transcription factor has been demonstrated to regulate endometrial gene expression patterns, with its reduced expression exerting detrimental effects on uterine receptivity and compromising embryo implantation potential (Elbaz et al., 2018).

The study findings indicate that human amniotic epithelial cells (hAECs) can mediate partial functional recovery of ovarian activity in premature ovarian failure via paracrine signaling pathways, with their therapeutic efficacy potentially mediated through the downregulation of transforming growth factor-β1 (TGF-β1) expression in stimulated CD4^+^ T lymphocytes from RSA patients (Yao et al., 2016; Motedayyen et al., 2018). Vitamin D has been shown to modulate the immune response at the fetomaternal interface and adequate vitamin D levels may play an important therapeutic role in RSA. (Sharif et al., 2018). Higher levels of vitamin D may trigger different anti-inflammatory functions including the function of T regulatory cells (Tregs) and/or increasing their numbers (Sakaki et al., 2005). Moreover, the inflammation caused by the excessive deposition of neutrophil extracellular traps (NETs) is an important factor in the pathogenesis of POI (Chen et al., 2023). However, the pathogenesis of RSA and POI is not well understood. The therapeutic effects and potential of these two medicines are still unclear and further exploration is necessary to determine whether they can effectively treat both diseases and whether they can be used clinically. Therefore, in order to understand the mechanism of comorbidity therapeutic measures have been proposed to clarify the diseases’ pathogenesis and to develop precise targeted therapies.

There are significant limitations in the current bioinformatics research on POI and RSA comorbidity genes. Firstly, most analyses rely on a single dataset and lack multi omics integration, resulting in insufficient robustness of the results. Secondly, it lacks clinical translational value and fails to systematically explore potential applications for diagnosis or treatment. Therefore, there is an urgent need to adopt more powerful computational biology methods to systematically elucidate its comorbidity mechanisms and explore clinical potential.

**FIGURE 1:**
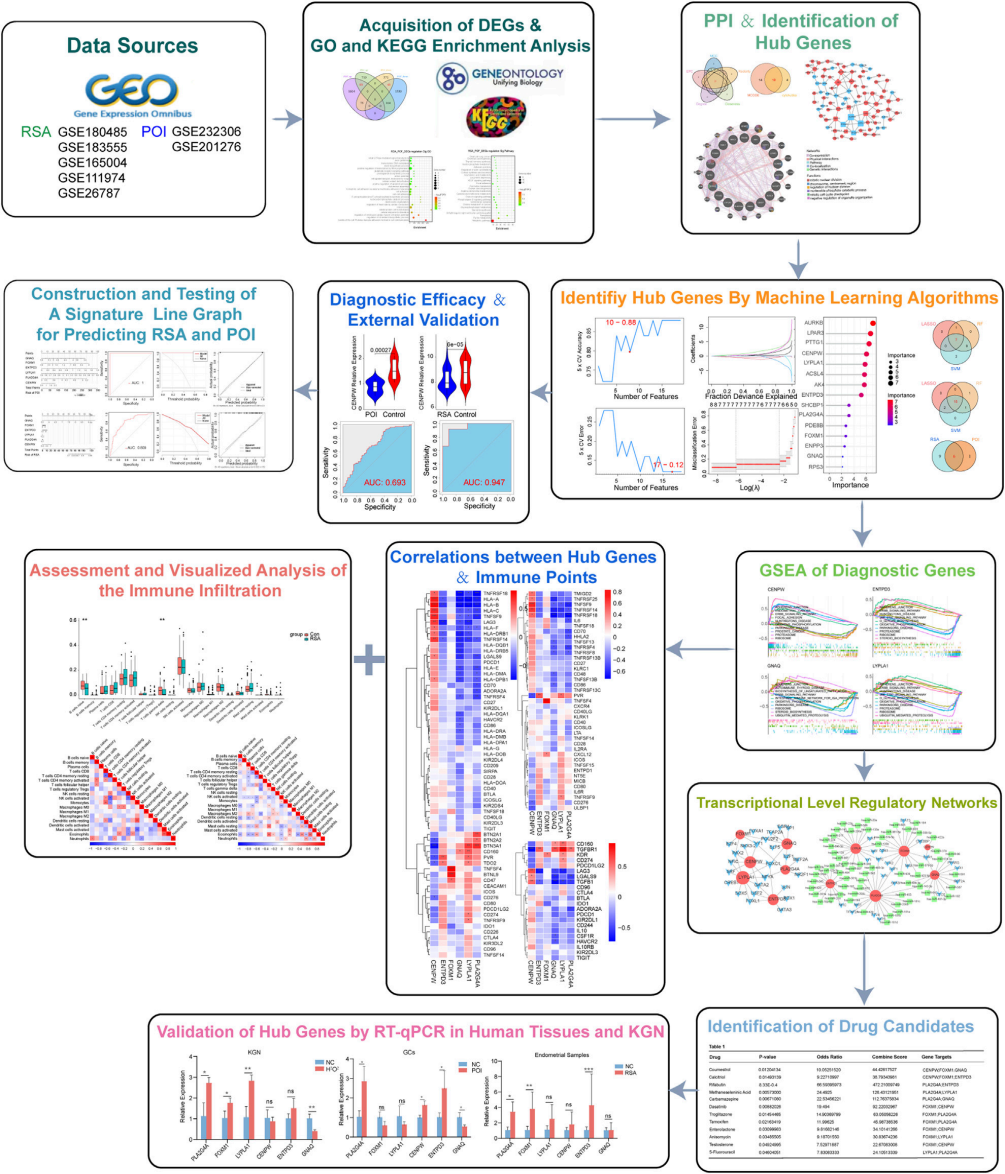
Flow diagram of the study.

In this study, we explored the hub genes between POI and RSA and investigated their diagnostic value and target enrichment. We collected RNA sequencing data from the GEO database on the granulosa cells of POI patients and the endometrial tissue of RSA patients and screened the hub genes associated with these two diseases. Function annotation, protein–protein interaction (PPI) network, transcription factor (TF)-gene regulatory network, microRNA (miRNA)-gene regulatory network, diagnostic histogram, and gene interaction network were constructed based on the DEGs and hub genes. A diagnostic prediction model for these two diseases is proposed, which provides a basis for further exploration of the relationships between immune cells and these diseases. In addition, drugs that may be effective against RSA and POI are predicted using drug target enrichment analysis. These studies provide new insights into common molecular mechanisms of disease and demonstrate the potential of diagnostic markers of disease. Our results suggest that the copathogenesis of RSA and POI is significantly positively correlated with CENPW, ENTPD3, FOXM1, GNAQ, LYPLA1, and PLA2G4A (Figure 1).

This study may improve fertility and pregnancy outcomes in many women and provide new insights into treatment strategies and the management of RSA and POI.

## 2 Materials and methods

### 2.1 Research object

Select 30 POI patients who underwent *in vitro* fertilization (IVF)/intracytoplasmic sperm injection (ICSI) assisted pregnancy at the Reproductive Medicine Center of the Fourth Affiliated Hospital of Jiangsu University in 2023 and 2024, and 10 patients who received treatment due to tubal and/or male factors during the same period, and collect granulosa cells (Supplementary Table 1). Select 15 patients who underwent abortion due to recurrent miscarriage at the Reproductive Medicine Center of the Fourth Affiliated Hospital of Jiangsu University in 2023 and 2024, and 10 patients who did not have a history of adverse pregnancy and childbirth during the same period, and collect tissue (Supplementary Table 2). Exclusion criteria: 1. Use of steroid drugs within the past 3 months; 2. Organic lesions of the uterus; 3. History of autoimmune diseases; 4. History of radiotherapy and chemotherapy.

### 2.2 Methods

#### 2.2.1 Data collection and preparation

The data sets related to POI and RSA were screened in the Gene Expression Omnibus database (GEO) (http://www.ncbi.nlm.nih.gov/geo/) since both diseases were designed in this study. For RSA, we used the keyword “RSA” or “endometrial tissue” to search gene expression profiles. Inclusion criteria were as follows (1): RSA patients and normal controls must be included in the profiles (2) endometrial tissue should be used for sequencing. Accordingly, we selected five datasets numbered GSE180485, GSE183555, GSE165004, GSE111974 and GSE26787. The RSA dataset is relatively small, and recurrent implantation failure (RIF) patients GSE111974 were included in the study cohort. The clinical background of this sample is highly similar to the RSA population (with no clear history of infertility, only recurrent miscarriage), and can be used as a 'parallel validation group' for the RSA cohort. Additionally, some RSA patients ultimately need to achieve pregnancy through IVF. The core function is to validate the biological pathways discovered by the RSA queue, rather than independently supporting the clinical conclusions of RSA. For POI, the eligibility criteria were as follows (1): the profiles must include normal controls and the POI patients, and (2) the sample source must be granulosa cells. So, we selected two datasets numbered GSE232306, GSE201276 (Supplementary Table 3).

#### 2.2.2 Differential gene expression analysis

We employed the “sva” R package to identify and construct surrogate variables for high-dimensional datasets, effectively removing batch effects. Following data preparation for each condition, we conducted a comparative analysis of RSA and POI datasets using the Linear Models for Microarray (LIMMA) package in R (version 4.1.2). Differentially expressed genes (DEGs) were calculated between disease and control groups with it. For RSA, the DEG threshold was set as P value <0.05. For POI, the P value to 0.05 were used to identify the DEGs. And finally use Principal Component Analysis (PCA) to check the batch effect before and after correction. Next, the results of the differential analysis for each group were displayed using volcano plots, with blue indicating low expression and red representing high expression. Using the Venn Diagram tool, we pinpointed key genes that were jointly upregulated and downregulated in RSA and POI patients, which may serve as potential biomarkers for these two diseases.

#### 2.2.3 GO and KEGG analysis and validation of key genes

Gene Ontology (GO) analysis and Kyoto Encyclopedia of Genes and Genomes (KEGG) pathway enrichment analysis (Sherman et al., 2022) were conducted on differentially expressed genes (DEGs) identified from the endometrial tissue of RSA patients and granulosa cells of POI patients. Utilizing the “clusterProfiler” package, these analyses aimed to elucidate the biological functions and associated pathways of the DEGs. GO analysis and KEGG enrichment analysis were statistically significant at P < 0.05. GO analysis included three components, biological process (BP), cellular component (CC), and molecular function (MF).

#### 2.2.4 Construction of protein-protein interaction network and hub genes selection

PPI networks play a crucial role in advancing functional biology research. Using the STRING database (version 11.5, available at https://string-db.org) (Szklarczyk et al., 2015), a PPI network for the shared DEGs was constructed.

The CytoHubba plugin algorithm was employed to identify hub genes within the PPI network using multiple topological analysis methods, including EPC, MCC, Radiality, Closeness, and Degree. Additionally, the online Hiplot platform (https://hiplot.com.cn/cloud-tool) was utilized to identify co-hub genes by intersecting the candidate genes derived from CytoHubba and MCODE analyses.

GeneMANIA finds other genes that are related to a set of input genes, using a very large set of functional association data (Franz et al., 2018). Association data include protein and genetic interactions, pathways, co-expression, co-localization and protein domain similarity. In this study, GeneMANIA was used to analyze the PPI networks of our signature genes.

#### 2.2.5 Identification of relevant transcription factors and TF-miRNAs regulatory network

Transcription factors control chromatin and transcription by means of identifying specific DNA sequences. Using the NetworkAnalyst 3.0 platform (https://www.networkanalyst.ca/) and *H. sapiens* data, to predicte potential transcription factors (TFs) for the hub genes by accessing the ENCODE database (Zhou et al., 2019). The TF-gene regulatory network was then mapped and visualized using Cytoscape.

#### 2.2.6 Feature selection by three well-established machine learning algorithms

Three machine learning algorithms: LASSO, Random Forest and SVM-RFE, were used to screen signature genes. The hub genes were initially assessed by least absolute shrinkage and selection operator (LASSO) Cox proportional hazards regression using the “glmnet” R package. The optimal model was selected by identifying the penalty parameter (λ) value associated with the minimum partial likelihood deviance, determined through 10-fold cross-validation. A random forest algorithm was used to rank the importance of marker genes associated with RSA. SVM-RFE was used to further screen for signature genes, preserve genes with high average rankings for subsequent analyses. Genes identified using LASSO, random forest and SVM-RFE were intersected to obtain our signature genes.

#### 2.2.7 Modeling and testing of two diseases diagnostic nomogram

Using the rms R package, we constructed a nomogram to aid in the diagnosis of RSA and POI. The risk score was determined by aggregating the expression-based risk scores of individual core genes, resulting in a total risk score. The diagnostic value of the nomogram for two diseases was assessed using decision tree, calibration and ROC curves. The expression level and diagnostic value of the obtained hub genes were constructed by receiver operating characteristic (ROC) curves and the area under the curve (AUC) with 95% confidence intervals (CI) to assess the levels of hub genes distinguishing on POI and RSA using R software.

#### 2.2.8 Gene set enrichment analysis

MSigDB (c2.cp.kegg_legacy.v2023.2.Hs.entrez0) was used to download gene sets. We used the ssGSEA function in the GSVA package to calculate the GSVA score for each gene set in different samples. Enrichplot was used to show the top five activating and inhibiting pathways for each gene in the two disease groups.

#### 2.2.9 Differences and correlation analysis of immune cell infiltration

CIBERSORTx is a computational tool designed to estimate the proportions of immune cell subpopulations within tissue samples by employing a deconvolution algorithm based on gene expression data (Newman et al., 2019). Gene expression profile data from both disease-affected and healthy individuals were uploaded to CIBERSORTx to evaluate the proportions of immune cell subpopulations in patients with POI and RSA.The LM22 signature matrix file, encompassing 22 immune cell components, served as the reference for immune cell quantification. Pearson correlation analysis was employed to assess the relationships between different immune cell types.

#### 2.2.10 The relationship of hub genes and immunity

The correlation between immune infiltrated cells and diagnostic target biomarkers was determined by Pearson correlations. And the correlation of hub gene expression with several immunoregulators were evaluated using Pearson’s correlation coefficients.

#### 2.2.11 Drug-targeted GSEA enrichment analysis

To identify key drugs targeting specific genes, we utilized the Drug Signature Database (DSigDB) available through the web server (https://maayanlab.cloud/Enrichr/). This database provides comprehensive drug-gene interaction information and facilitates gene set enrichment analysis (GSEA). We identified drug candidates for the possible treatment of two diseases based on a statistical threshold of p-value <0.05 and drug targets ≥ 2.

#### 2.2.12 Genetic validation

The KGN cell line involved in this study was purchased from the cell bank of the Chinese Academy of Sciences. Cultivate in a DMEM/F12 medium containing 10% fetal bovine serum and 1% penicillin streptomycin at 37 °C and 5% CO2. Inoculate KGN cells with 6-well plates and add Cytoxan (CTX) to construct an oxidative stress model (Zhang et al., 2025). According to the instructions, TRIzol reagent (Invitrogen, USA) was used to lyse cells and extract total RNA. HiScript II Q RT SuperMix (Nanjing Novozan Biotechnology Co., Ltd.) was used to reverse transcribe the total RNA to obtain cDNA. ChamQ Universal SYBR qPCR Master Mix (Nanjing Novozan Biotechnology Co., Ltd.) was used for RT-qPCR. GAPDH is used as an endogenous reference. All primers were designed and synthesized by Shanghai Shenggong Biotechnology Co., Ltd. Related gene RT qPCR amplification primers can be found in Supplementary Table 1.

#### 2.2.13 Statistical analyses

All data are expressed as mean ± S.E.M, all follow a normal distribution. Statistical analysis was performed using GraphPad Prism 9.0 software. Differences between two independent groups were calculated using unpaired Student’s t-test. Statistical significance was set at P < 0.05; *<0.05, **<0.01, and ***<0.001.

## 3 Results

### 3.1 Identification of differentially expressed genes and functional enrichment analysis of the gene set

**FIGURE 2:**
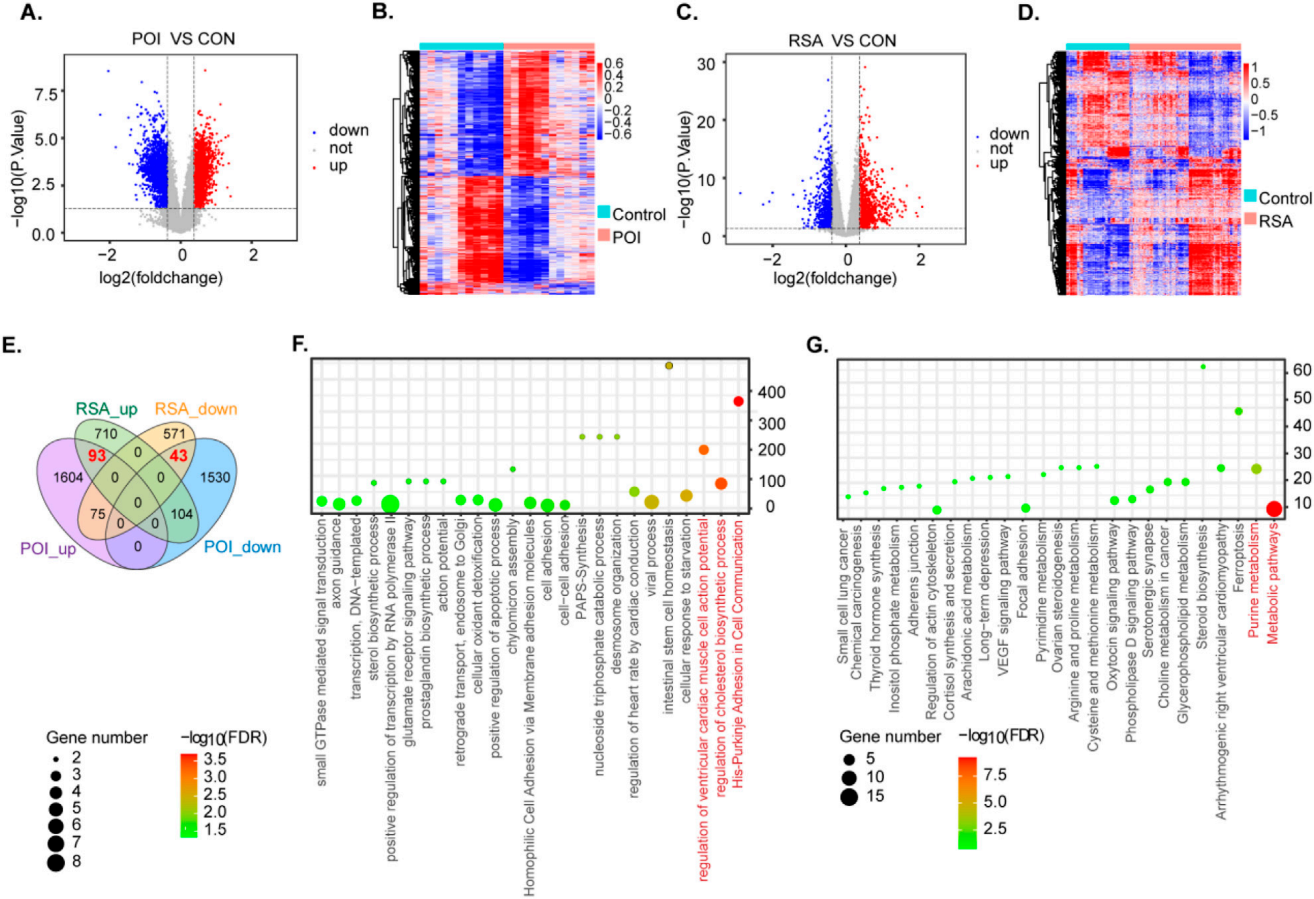
Identification and functional enrichment analysis of differentially expressed genes in two diseases. **(A,B)** DEG heatmap and volcano plot in POI group. **(C,D)** DEG heatmap and volcano plot in RSA group. **(E)** The intersection of DEGS of two diseases. **(F,G)** GO enrichment analysis and KEGG enrichment analysis of POI-RSA-DEGs DEG. (P < 0.05).

We obtained datasets from the GEO database and DEGs using the limma tool overlapping genes among these microarray datasets. In the POI datasets, we identified 3449 DEGs including 1782 upregulated genes and 1,677 downregulated genes (P < 0.05, |log2FC| > 2) (Figure 2A). For the RSA datasets, we detected 1,596 DEGs comprising 686 upregulated genes and 910 downregulated genes (P < 0.05, |log2FC| > 2) (Figure 2C). The DEGs from both groups are represented with heatmaps (Figures 2B,D). Through a comparison of these two datasets we identified 136 DEGs between POI and RSA, comprising 93 upregulated genes and 43 downregulated genes. These DEGs may contribute to the onset and progression of POI and RSA (Figure 2E). Due to the different sources of the dataset, when using PCA to check the batch effects before and after correction, it can be seen that there is a significant batch effect before batch removal, but there is no significant batch effect after batch removal (Supplementary Figure 1).

To delve deeper into potential molecular mechanisms associated with RSA and POI comorbidity we integrated GO and KEGG pathway annotations to characterize the 136 DEGs obtained in the previous screening step. Notably, among the enriched GO terms we identified several significantly enriched biological process pathways, such as regulation of cholesterol biosynthetic processes (Figure 2F). With respect to KEGG pathways, examples include metabolic pathways and purine metabolism (Figure 2G).

### 3.2 Protein-protein interactions and identification of hub genes using MCODE and CytoHubba

**FIGURE 3:**
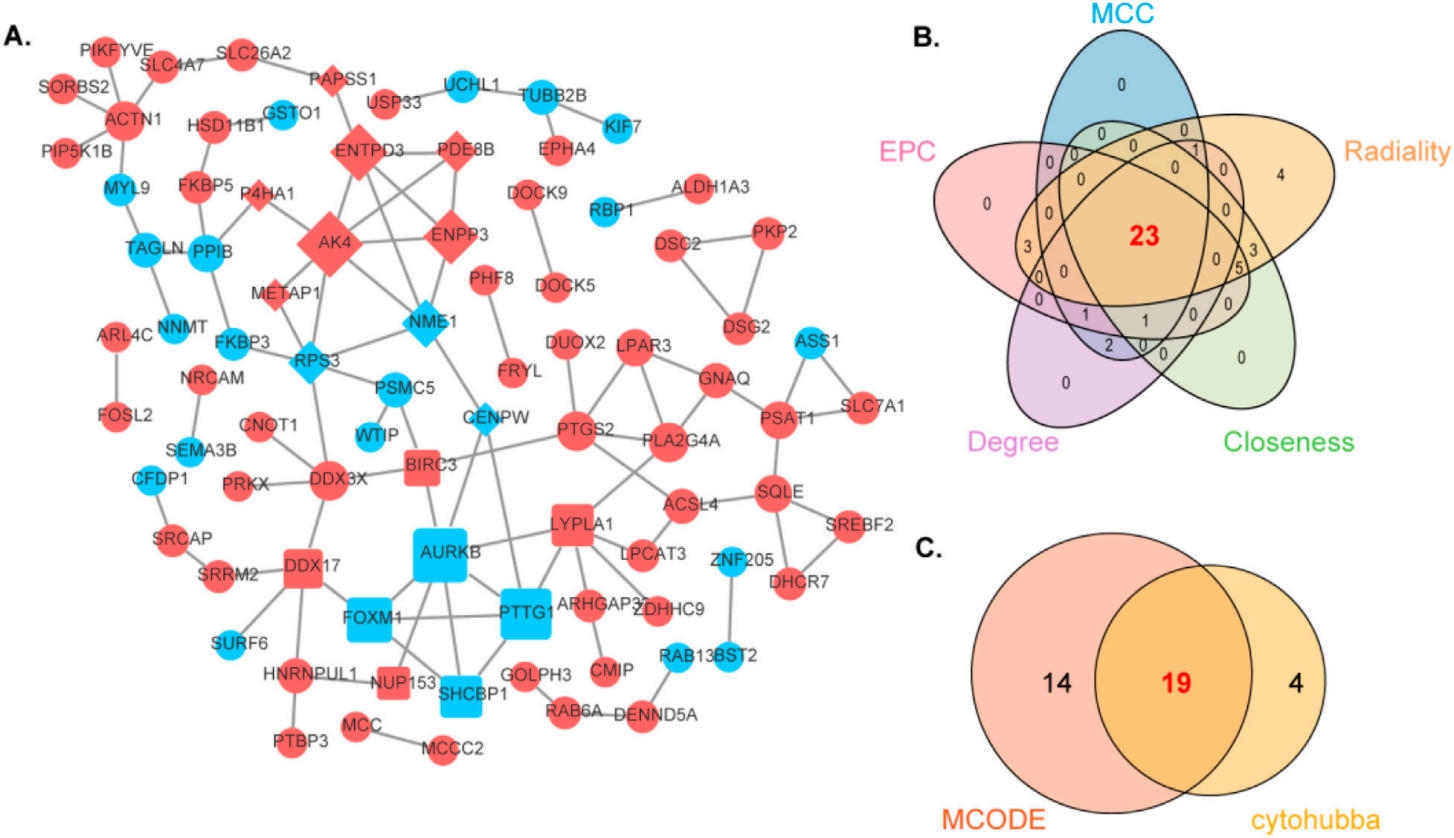
Analyzing the protein-protein interaction network of DEGs through MCODE and CytoHubba and further analyzing hub genes. **(A)** The protein-protein interaction network of POF-RSA-DEGs. **(B)** Intersection of five cytohubba algorithms. **(C)** Intersection of MCODE and Cytohubba.

We integrated the shared DEGs into a PPI network to explore potential interactions (Figure 3A). Utilizing the CytoHubba plug-in within Cytoscape we conducted topological analysis employing five algorithms to pinpoint hub genes. A total of 23 common DEGs were subsequently identified through the intersection of CytoHubba and visualized via Venn diagrams. We then overlapped the candidate DEGs from CytoHubba with the 23 shared DEGs from MCODE analysis, revealing 19 shared genes that are illustrated through Venn diagrams (Figures 3B,C).

To delve deeper, GeneMANIA biological function analysis was implemented to explore genes with functions similar to those of the aforementioned 19 shared DEGs and to elucidate the interactive functional association network among genes. The results revealed 73.53% co-expression between genes, 21.49% physical interactions, 3.27% pathway interactions, 1.71% co-localization, and 0.00% genetic interactions (Supplementary Figure 2). The functions attributed to these genes were primarily linked to immune response-regulating cell surface receptor signaling pathways involved in mitotic nuclear division, chromosome centromeric region, regulation of nuclear division, nucleoside phosphate catabolic processes, mitotic cell cycle checkpoints, and negative regulation of organelle organization.

### 3.3 Identification of potential shared diagnostic genes based on machine learning algorithms and validation of hub genes via RT‒PCR in human tissues and KGN

**FIGURE 4:**
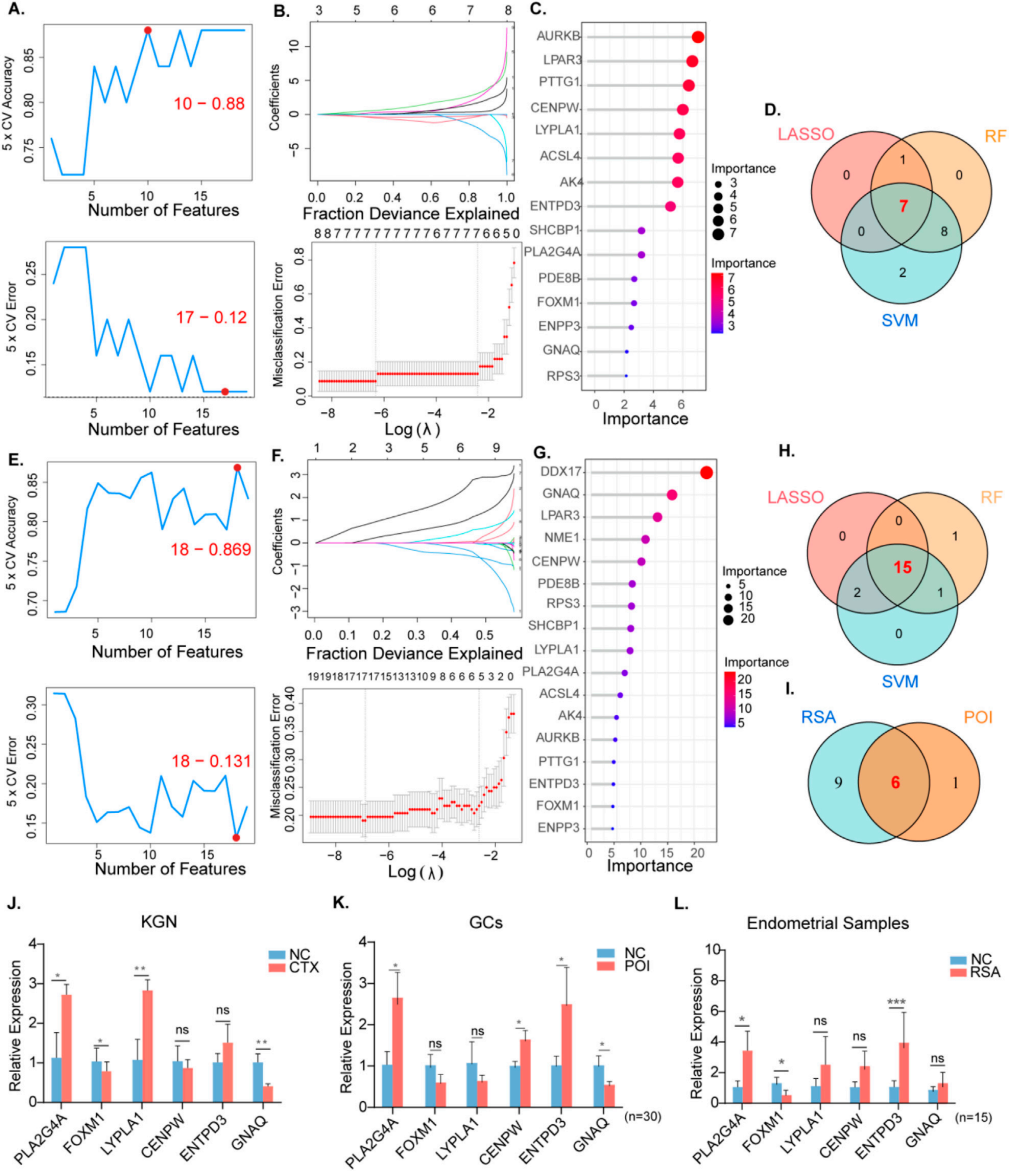
The screening of candidate POI and RSA diagnostic genes using three machine learning algorithms.Validation of RT qPCR in human tissues and KGN cells. POI: **(A)** Biomarker signature gene expression validation via support vector machine recursive feature elimination (SVM–RFE) algorithm selection. **(B)** Adjustment of feature selection in the minimum absolute shrinkage and selection operator model (LASSO). **(C)** The top 20 relatively important genes. **(D)** Three algorithmic Venn diagram screening genes. **(E-H)** For RSA, same as before. **(I)** Intersect the genes obtained from the above intersection again. **(J)** Expression levels of six hub genes in normal KGN and CTX models **(K)** Expression levels of hub genes in granulosa cells of normal and POI patients. (n = 30) **(L)** Expression levels of hub genes in endometrial tissues of normal and RSA patients. (n = 15).

To screen for candidate diagnostic gene targets with distinct characteristics, three machine learning algorithms (LASSO, SVM-RFE, and random forest) were employed on 19 shared genes identified previously. In the POI group, regarding SVM-RFE, the classifier error was minimized with 17 features (Figure 4A). By analyzing LASSO coefficient profiles and selecting the optimal tuning parameter, λ was set at 0.001792538 for POI, resulting in the discovery of eight genes with nonzero coefficients (Figure 4B). Additionally, using the random forest algorithm 15 feature genes with a relative importance score of two were distinguished (Figure 4C). Through the overlap of these three algorithms seven shared biomarkers were subsequently established (Figure 4D).

Similarly, for the RSA group, 15 featured genes were extracted when a subset of 18 hub genes was revealed using the SVM-RFE algorithm (Figure 4E). The top 17 genes based on the importance scale are displayed in Figure 4. F, where 17 genes with importance >4 were chosen as the RSA result. The λ value was subsequently determined to be 0.0010173 using the LASSO algorithm (Figure 4GH). By intersecting the machine learning results between the RSA and POI groups, six shared diagnostic genes were identified: CENPW, ENTPD3, FOXM1, GNAQ, LYPLA1, and PLA2G4A (Figure 4I).

RT-PCR was conducted on follicular fluid-derived granulosa cells from normal women and POI patients, as well as on endometrial tissues from healthy individuals and RSA patients. This verified the gene expression levels of the six diagnostic biomarkers. Compared with the data analysis, our results indicated that the expression patterns of the six genes in KGN cells were consistent with the aforementioned analysis outcomes (Figure 3J). In the granulosa cells of POI patients the expression of PLA2G4A and CENPW was upregulated, while the expression of GNAQ was downregulated (Figure 4K). In the decidual tissue of RSA patients, with the exception of CENPW and GNAQ, the results were in accordance with the data analysis (Figure 4L).

### 3.4 Construction of transcriptional-level regulatory networks and testing of a signature gene-based line graph for prediction

To elucidate the biological processes underlying disease pathogenesis we examined the interplay between integrated transcription factors, miRNAs, and hub genes. In this investigation, we utilized the Networkanalyst platform, which incorporates data from ENCODE (https://www.encodeproject.org/), and RegNetwork (http://www.regnetworkweb.org) databases to construct the TF‒gene interaction network and TF‒miRNA coregulatory networks.

**FIGURE 5:**
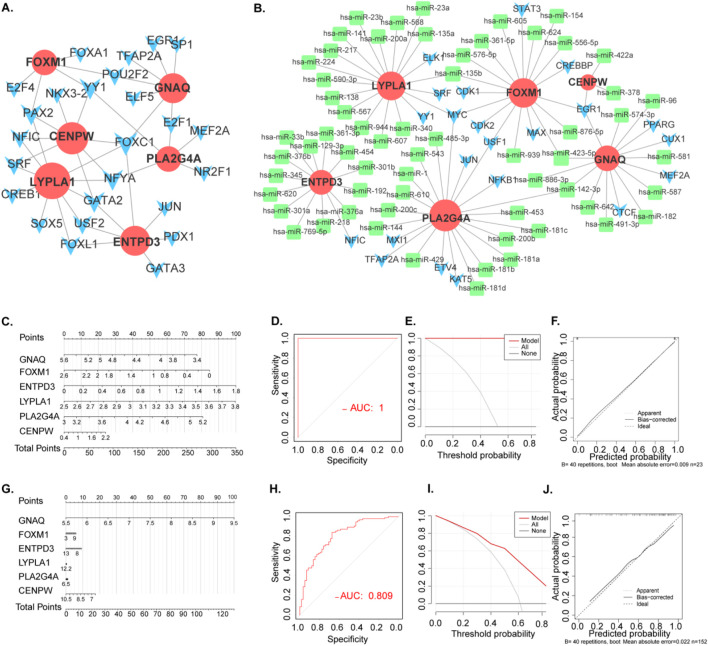
Transcriptional Level Regulatory Networks and construction and validation of POI and RSA diagnostic column line graph model. **(A)** PPI network. The nodes represent proteins, the edges represent their interaction. **(B)** TF-gene interaction network and TF-miRNA co-regulatory networks.Green rectangle: hub gene related mirRNA. Blue arrow: TF **(C)** Column line graphs were used to predict the occurrence of POI. **(D)** Calibration curves assessed the predictive power of the column line graph model. **(E)** DCA curves were used to assess the clinical value of the column line graph model. **(F)** ROC curves assessed the clinical value of the column line graph model. **(G)** Column line graphs were used to predict the occurrence of RSA. **(H)** Calibration curves assessed the predictive power of the column line graph model. **(I)** DCA curves were used to assess the clinical value of the column line graph model. **(J)** ROC curves assessed the clinical value of the column line graph model.

The TF-gene network involved 25 transcription factors, six hub genes, and 41 edges, delineating their interactions. Concurrently, the TF‒miRNA coregulatory network revealed a total of 91 edges, with 61 miRNAs and 22 TF genes engaging in regulatory associations with the six hub genes (Figures 5A,B). This detailed analysis illuminates the intricate relationships among transcription factors, miRNAs, and hub genes, enriching our comprehension of the regulatory mechanisms driving disease pathogenesis.

We employed diagnostic genes (CENPW, ENTPD3, FOXM1, GNAQ, LYPLA1, and PLA2G4A) and the “Rms” R package to develop diagnostic histogram models for RSA and POI. The predictive power of these models was evaluated through calibration curves (Figure 5C). Notably, the calibration curves exhibited minimal variances between the true and predicted disease risks, indicating the high accuracy of the bar graph model (Figure 5D).

DCA indicated potential benefits for POI patients utilizing such nomograms (Figure 5E). Furthermore, the model’s validity was confirmed via ROC curve analysis (Figure 5F). These analyses were similarly conducted for RSA (Figures 5G–J), providing a robust framework for predicting both RSA and POI basis of the signature gene expression.

### 3.5 Diagnostic efficacy and external validation of signature genes in POI and RSA

**FIGURE 6:**
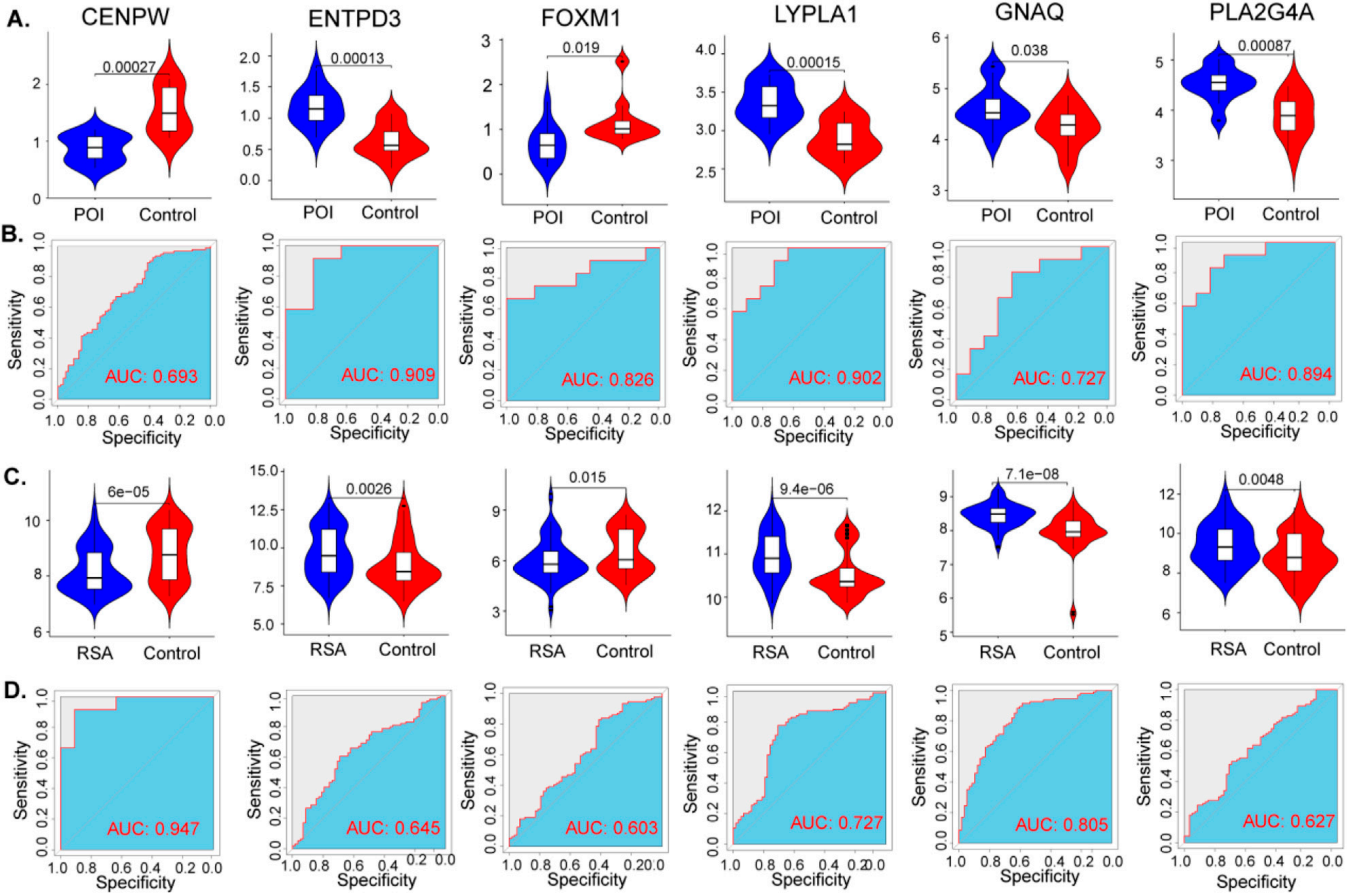
Diagnostic efficacy of the target genes in the prediction of POI and RSA. **(A)** The violin plot shows the mRNA expression of six hub genes in the disease and control groups in the datasets of POI. **(B)** ROC curves estimating the diagnostic performance of the six hub genes in the datasets of POI. **(C,D)** Same as before, the violin plot and ROC curves of RSA.

The expression levels of the six hub genes were statistically significant in both the POI and RSA samples (Figures 6 A,C). The diagnostic performance of these signature genes was estimated for predicting POI using AUC values: 0.693 for CENPW, 0.909 for ENTPD3, 0.826 for FOXM1, 0.902 for LYPLA1, 0.727 for GNAQ, and 0.894 for PLA2G4A. For the prediction of RSA the AUC values were as follows: 0.947 for CENPW, 0.645 for ENTPD3, 0.603 for FOXM1, 0.727 for LYPLA1, 0.805 for GNAQ, and 0.627 for PLA2G4A (Figures 6B,D).

These findings highlight the potential diagnostic efficacy of the identified signature genes in distinguishing between POI and RSA. The AUC values signify the discriminatory power of these genes and their ability to serve as reliable markers for these conditions.

### 3.6 GSEA of diagnostic genes

We subsequently conducted GSEA on the diagnostic genes in both the RSA and POI datasets. The top five upregulated and downregulated pathways were visualized using the “GSEA” package.

In both the POI and RSA datasets, CENPW, GNAQ, LYPLA1, and PLA2G4A were found to be associated with the ribosome pathway. ENTPD3 is linked to the oxidative phosphorylation, ribosome, and steroid biosynthesis pathways. FOXM1 is involved in base excision repair, the cell cycle, and DNA replication pathways (Supplementary Figures 3A,B).

These results provide insights into the potential biological mechanisms and pathways associated with the diagnostic genes in RSA and POI, shedding light on their functional roles and implications in disease pathogenesis.

### 3.7 Assessment and visual analysis of immune infiltration

**FIGURE 7:**
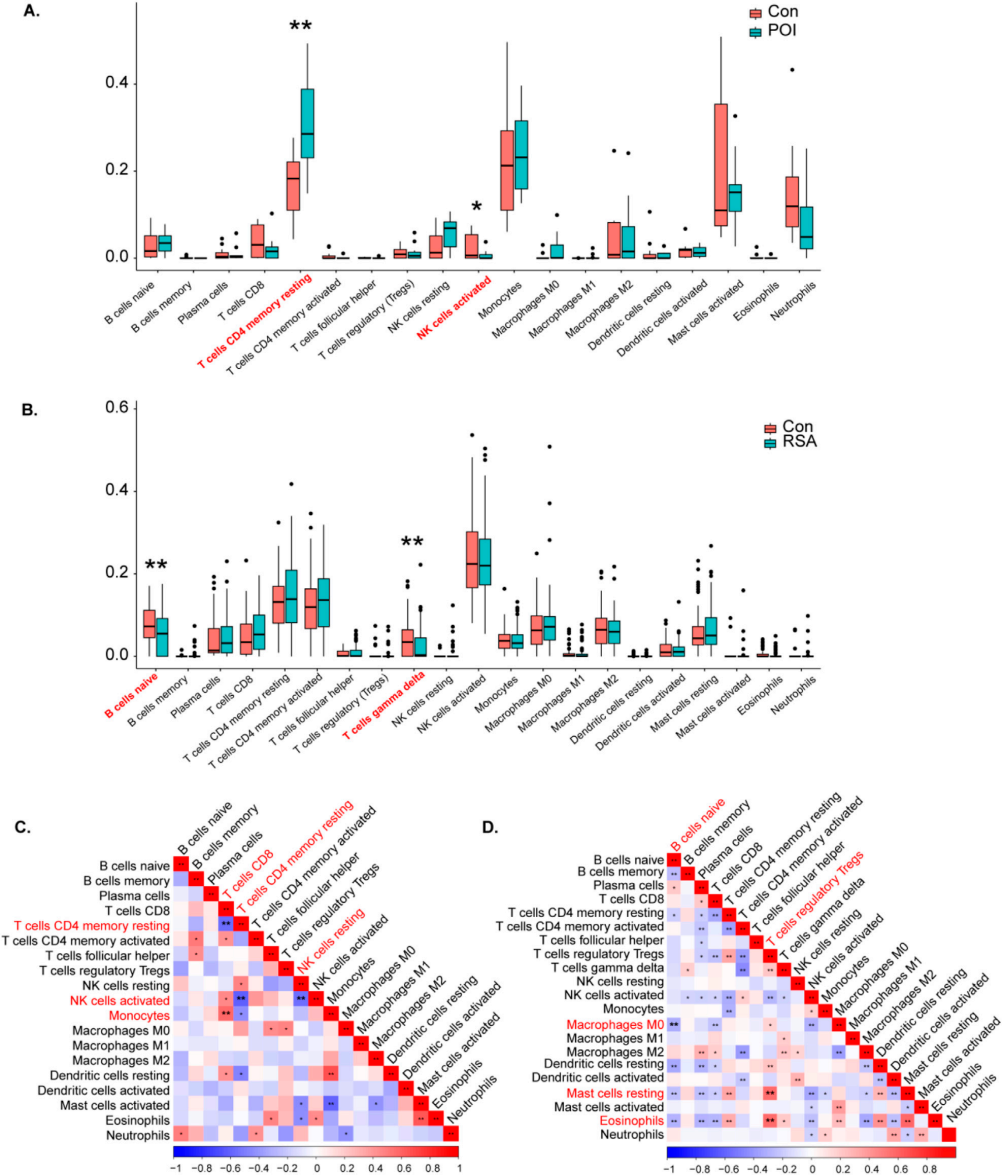
Analysis of POI and RSA immune cells. **(A, B)** Violin diagram indicated that the POI and RSA group exhibited a significantly different type of immune cell. **(C)** The correlation between immune cells in POI. **(D)** The correlation between immune cells in RSA.

The abundances of immune cells in different groups were analyzed using CIBERSORT, with the proportions of 19 immune cells depicted in bar plots. Notable findings include the following:

POI Samples:T cells CD4 memory resting, monocytes, mast cells activated, and activated neutrophils were prominent in POI samples (Supplementary Figures 4A,B). Compared with those in the control samples, increases in the numbers of resting memory T cells and monocytes werer observed in the POI samples (Figure 7A). Further analysis revealed significant negative correlations between immune cell types in the POI datasets, such as memory T cells CD4 resting with CD8, and NK cells activated with resting memory CD4 T cells and resting NK cells. Monocytes were significantly positively correlated with CD8 T cells (Figure 7C).

RSA Samples:

Activated NK cells, resting memory CD4 T cells, and activated memory CD4 T cells were predominant in the RSA samples (Supplementary Figure 3 C, D). Compared with those in control samples reductions in naive B cells and gamma delta T cells were observed in RSA samples (Figure 7B). A correlation analysis of the RSA datasets revealed significant negative correlations between naive B cells, resting B cells, and M0 macrophages. In addition, T-cell regulatory Tregs were significantly positively correlated with resting mast cells and eosinophils (Figure 7D).

These findings offer insights into the immune cell composition and interactions within POI and RSA samples, providing valuable information on immune infiltration patterns associated with these conditions.

### 3.8 Correlations between hub genes and immune points

**FIGURE 8:**
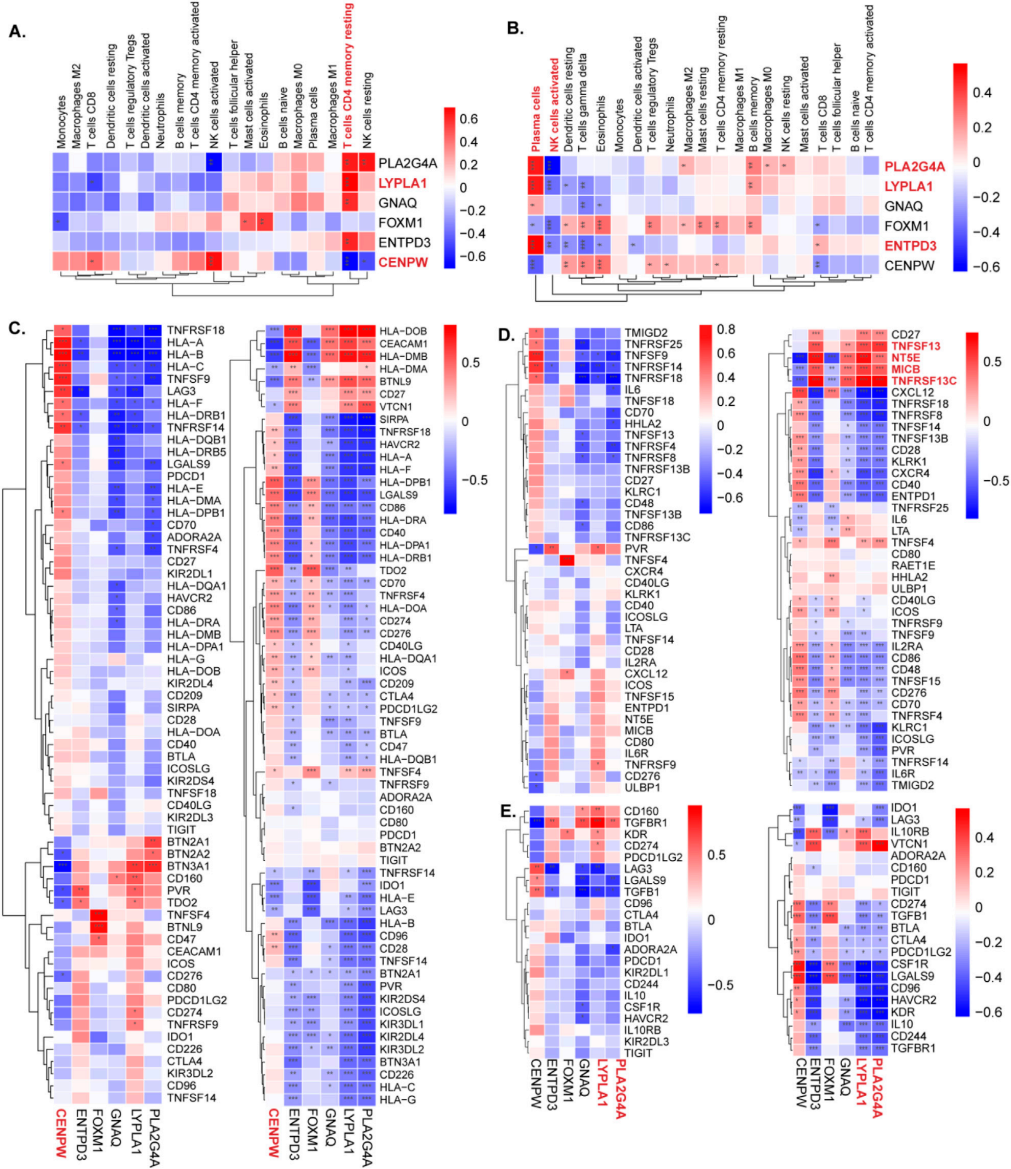
The relationship between diagnostic genes and immune cells and immune genes. **(A,B)** The expression of immune cells of CENPW, ENTPD3, FOXM1, GNAQ, LYPLA1, and PLA2G4A in the POI and RSA groups was detected separately. **(C)** The correlation between hub genes and immune checkpoints in the POI and RSA groups. **(D)** The correlation between hub genes and immune activate in the POI and RSA groups. **(E)** The correlation between hub genes and immune inhibit in the POI and RSA groups.

The correlation between immune cell infiltration and the shared hub genes was examined to elucidate differences in the immune microenvironment between diseased patients and healthy controls. Key findings include the following:

POI datasets: The most significant positive correlation with resting memory CD4 T cells was observed for most biomarkers, except CENPW, which displayed a negative correlation (Figure 8A).

RSA datasets: ENTPD3, LYPLA1, and PLA2G4A exhibited positive correlations with plasma cells in RSA samples. Additionally, PLA2G4A was negatively correlated with activated NK cells (Figure 8B).

Moreover, the TISIDB database was used to acquire immune checkpoint, immune activation point, and immune inhibition point data.

For both diseases: CENPW was positively correlated with most immune checkpoint points. PLA2G4A, ENTPD3, and LYPLA1 exhibited strong positive correlations with immune agonists specific to RSA, such as TNFSF13, NT5E, MICB, and TNFRSF13C. With respect to immune inhibition points, PLA2G4A and LYPLA1 displayed inverse correlations with most points (Figures 8 C–H).

These analyses provide insights into the relationships between hub genes and immune cell infiltration, shedding light on the complex interplay between genetic markers and the immune microenvironment in POI and RSA conditions.

### 3.9 Identification of drug candidates

**FIGURE 9:**
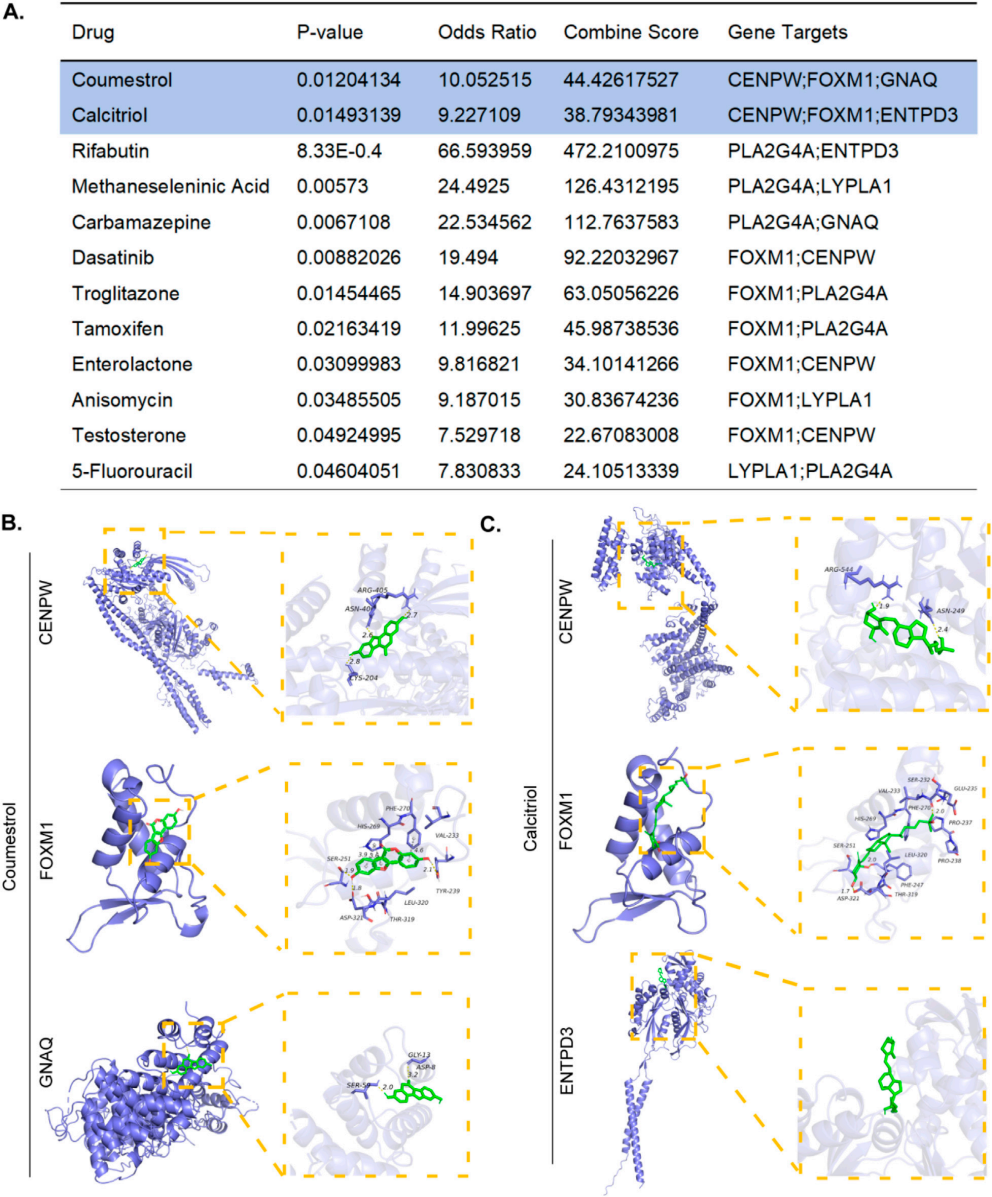
Prediction of candidate drugs. **(A)** Top 12 therapeutic drug candidates generated. **(B)** The molecular docking simulations of Coumestrol to CENPW/FOXM1/GNAQ. **(C)** The molecular docking simulations of Calcitriol to CENPW/FOXM1/ENTPD3.

To facilitate the development of therapeutics for RSA and POI, we performed targeted drug enrichment analysis on the basis of six diagnostic genes and identified 12 drug candidates (Figure 9A). Coumestrol can target three hub genes (CENPW, FOXM1, GNAQ), whereas Calcitriol could target three different hub genes. The remaining 10 drug candidates (Rifabutin, Methaneseleninic Acid, Carbamazepine, Dasatinib, Troglitazone, Tamoxifen, Enterolactone, Anisomycin, Testosterone, 5-Fluorouracil) could target two different hub genes.We speculate that these 12 drugs may exert therapeutic effects on POI and RSA by targeting proteins encoded by the identified key genes. Using molecular docking simulations to elucidate the therapeutic mechanisms of top2 drugs. The binding energies of CENPW, FOXM1, and GNAQ docked with Coumestrol are −8.2, −7.18, and −6.0 (kcal/mol), respectively. The binding energies of CENPW, FOXM1, and ENTPD3 docked with Calcitriol are −8.6, −6.83, and −7.1 (kcal/mol), respectively. Hydrogen bonding is a key molecular interaction force that stabilizes ligand receptor complexes and ensures binding specificity. Among the six molecular docking sites, except for the docking site between Calcitriol and ENTPD3 where no hydrogen bonds were present, all other docking sites had hydrogen bonds present (Figure 9B,C).

## 4 Discussion

POI exhibits a diverse clinical spectrum and serious health consequences, such as RSA, highlighting the need to explore shared pathogenesis between these diseases. Using MCODE, CytoHubba, and three machine learning methods, we identified a common gene set and six diagnostic genes—CENPW, ENTPD3, FOXM1, GNAQ, LYPLA1, and PLA2G4A—significantly linked to both POI and RSA. Further immune infiltration and checkpoint analyses revealed distinct patterns of abnormal immune activation associated with these hub genes. Our findings provide new clinical insights and potential targeted strategies for diagnosing and treating POI and RSA.

In screening 93 significantly upregulated DEGs and 43 significantly downregulated DEGs from seven datasets, subsequent GO enrichment analysis highlighted pathways such as positive regulation of transcription by RNA polymerase II and metabolic pathways. KEGG enrichment revealed that the DEGs were predominantly involved in pathways such as metabolic pathways and purine metabolism, suggesting a potential close association with the progression of POI and RSA.

CENPW, a key element of the CENPA-NAC (nucleosome-associated) complex, critically contributes to kinetochore assembly, mitotic progression control, and faithful chromosome segregation (as suggested by sequence homology) (Nishino et al., 2012). As a key regulator of kinetochore assembly and function, CENPW is essential for forming proper centromere-microtubule attachments during oocyte maturation. It ensures normal spindle formation and chromosome arrangement, supporting oocyte development progression from mid to late stages (Wang et al., 2020). Major pathological mechanisms of female infertility include disrupted oocyte meiotic maturation and aberrant chromosome segregation. Meanwhile, ENTPD3—characterized by threefold greater catalytic activity toward ATP than ADP hydrolysis—is involved in breast cancer-driving molecular pathways (Li et al., 2019). As a key transcriptional regulator, FOXM1 controls cell cycle genes involved in DNA replication and mitosis. It acts as a hub gene in POI pathogenesis, regulating proliferation, self-renewal, and tumor development (Liu D. et al., 2023; Liao et al., 2018). FOXM1 is essential for human stromal cell decidualization. Its uterine-specific deletion causes localized decidualization defects, impairing stromal cell mitosis and increasing polyploidy at the implantation site (Gao et al., 2015). GNAQ functions as a modulator or transducer in multiple transmembrane signaling pathways. Studies have demonstrated that overexpression of GNAQ can exert antioxidant and cytoprotective effects, counteracting aging processes driven by the accumulation of reactive oxygen species (ROS) (Sun et al., 2020). LYPLA1, which functions as a palmitoyl thioesterase, catalyzes protein depalmitoylation and can depalmitoylate GPCPD1, leading to mitochondrial autophagy and promoting tumor growth and metastasis in triple-negative breast cancer (TNBC) patients (Liu Y. et al., 2023). Current research indicates high LYPLA1 expression in malignant cervical cancer tissues (Kwon et al., 2023). PLA2G4A is a key enzyme in membrane lipid remodeling and lipid mediator biosynthesis during inflammation, and triggers prostanoid production essential for embryo implantation and parturition (Jin et al., 2023). Inhibiting PLA2G4A may improve placental perfusion and lower miscarriage risk by modulating prostaglandins (Johansen et al., 2000). As a specific necroptosis gene linked to POI, PLA2G4A expression is positively correlated with this condition (Ge et al., 2014).These findings support the identification of diagnostic genes related to POI and RSA, offering potential targets for investigating the molecular mechanisms underlying the progression of these diseases.

To explore the pathogenic associations and mechanisms between POI and RSA further, we conducted GSEA on the six diagnostic genes within the two disease groups. CENPW, GNAQ, LYPLA1, and PLA2G4A are linked to the ribosome pathway. In previous studies on spontaneous abortion (SA), Mendelian analysis revealed that GNAQ is closely related to the disease and is enriched in the ribosome pathway (Xiang et al., 2025). Whereas ENTPD3 is associated with the oxidative phosphorylation, ribosome, and steroid biosynthesis pathways. FOXM1 participates in base excision repair, cell cycle, and DNA replication pathways. Its collaboration with the androgen receptor (AR) promotes DNA synthesis and cell proliferation (Liu et al., 2014), while inherent differences in FOXM1 signaling contribute to varied responses to cell cycle therapy (Jawwad et al., 2025). FOXM1 is also established as a DNA repair gene in lung adenocarcinoma (Yang et al., 2020). Meanwhile, mitochondrial ribosomes are essential for ovarian function (Kline et al., 2022), and homozygous missense variants in the NOP14 gene—critical in 18S rRNA processing and 40S ribosome assembly—cause ribosome defects linked to RSA (Suzuki et al., 2018). Subsequent ROC analysis indicated that all central genes have potential diagnostic value in the clinical management of POI and RSA.

To validate the clinical diagnostic value of these six genes (including PLA2G4A and FOXM1), we analyzed KGN cells, granulosa cells from POI patients, and decidual tissue from RSA patients. Potential confounders such as age and BMI were controlled during sample selection. The expression patterns of these genes were consistent with dataset findings, supporting their potential diagnostic relevance for POI and RSA. Due to the difficulty of obtaining clinical samples, it is expected to expand the sample size in subsequent studies to support the results.

The immune cell composition in POI and RSA samples reveals distinct immunological profiles: POI is marked by resting CD4 memory T cells and activated NK cells, while RSA features naive B cells and activated γδ T cells, indicating condition-specific immune mechanisms. Elevated B-cell counts correlate with higher antiphospholipid antibody levels in the Antiphospholipid Antibody Syndrome (APS), suggesting B-cell involvement in the immune dysregulation of RSA (Youinou and Renaudineau, 2004). The case analysis of a woman with a history of RSA and infertility, showing low levels of memory B-cells, suggests a potential association between abnormal B cell subsets and reproductive issues (Sung et al., 2016). Additionally, activated γδ T cells increase during pregnancy and appear protective (Mincheva-Nilsson et al., 1994; Meeusen et al., 1993), whereas their decrease in RSA patients suggests a negative correlation with the condition. These immune characteristics enable precise patient stratification and offer direct therapeutic targets. CENPW, a core cell cycle regulator, is closely associated with cell proliferation (Nishino et al., 2012). We found that it is positively correlated with most immune checkpoints in both diseases. Studies have shown that rapidly proliferating cells, including tumor cells and activated immune cells, typically upregulate immune checkpoint molecules as a feedback self-protection mechanism to prevent excessive immune responses from causing self damage (Sandberg et al., 2008). The high expression of PLA2G4A directly indicates a strong inflammatory response (Leslie, 2015). TNFSF13 (April) and its receptor TNFRSF13C (BAFFR) are crucial for B cell activation, differentiation, and survival. Their co-upregulation indicates abnormal local B cell responses, potentially leading to autoantibodies against trophoblast or embryonic antigens (Vincent et al., 2013). Therefore, these hub genes likely form a complex immune network rather than functioning alone. Correlations between hub genes and specific immune cells or markers in POI and RSA patients reveal potential molecular drivers of these diseases.

Based on the drug targeted enrichment results, we found interesting aspects of coumestrol and calcitriol. Coumestrol, which acts as an estrogen receptor agonist (Jiang et al., 2013). In POI, hormone replacement therapy (HRT) should be considered as a physiological substitute for estrogen and progesterone The effect of coumarin may be similar to estrogen in HRT, thereby slowing down POI (Ishizuka, 2021). As the active hormone form of vitamin D, calcitriol may be an effective therapeutic approach for managing reproductive disorders by simultaneously regulating immune function and regulating endocrine pathways that are crucial for optimal reproductive health (Gonçalves et al., 2018; Kebapcilar et al., 2013). This study identified candidate drugs for POI and RSA via bioinformatics analysis, offering new treatment directions. However, translating these predictions into clinical use remains complex and long-term. Future efforts should establish a multi-index evaluation system to prioritize the most promising compounds, followed by *in vitro* and *in vivo* validation, as well as clinical cohort studies.

This study has certain limitations: the complexity of *in vitro* fertilization data regarding hormonal effects on infertility, a small sample size limiting external validation of hub genes, and potential bias in immune infiltration analysis when applying CIBERSORTx to homogeneous cell samples. Nevertheless, the findings offer valuable insights. Future research should expand datasets for robustness, perform prospective clinical validation, and extend molecular investigations beyond the transcriptome.

In conclusion, this study elucidates the intricate immune cell profiles of patients with POI and RSA, revealing promising biomarkers and pathways for further investigation. By addressing these limitations and building upon the identified diagnostic genes, including CENPW, ENTPD3, FOXM1, GNAQ, LYPLA1, and PLA2G4A, we pave the way for innovative approaches to understanding and potentially treating POI and RSA. This study sets the stage for collaborative efforts toward improving outcomes for individuals affected by these reproductive disorders.

## Data Availability

The datasets presented in this study can be found in online repositories. The names of the repository/repositories and accession number(s) can be found in the article/Supplementary Material.
