# Supplementary material for: Immune-molecular nexus in reproductive disorders: mechanisms linking POI and RSA
Source: Front Genet. 2025 Sep 29;16:1652519. doi: 10.3389/fgene.2025.1652519 (PMC12515496; doi:10.3389/fgene.2025.1652519)
Supplement: Supplementary file 1 [file Image1.pdf]

Supplementary material

Supplementary Figure 1.

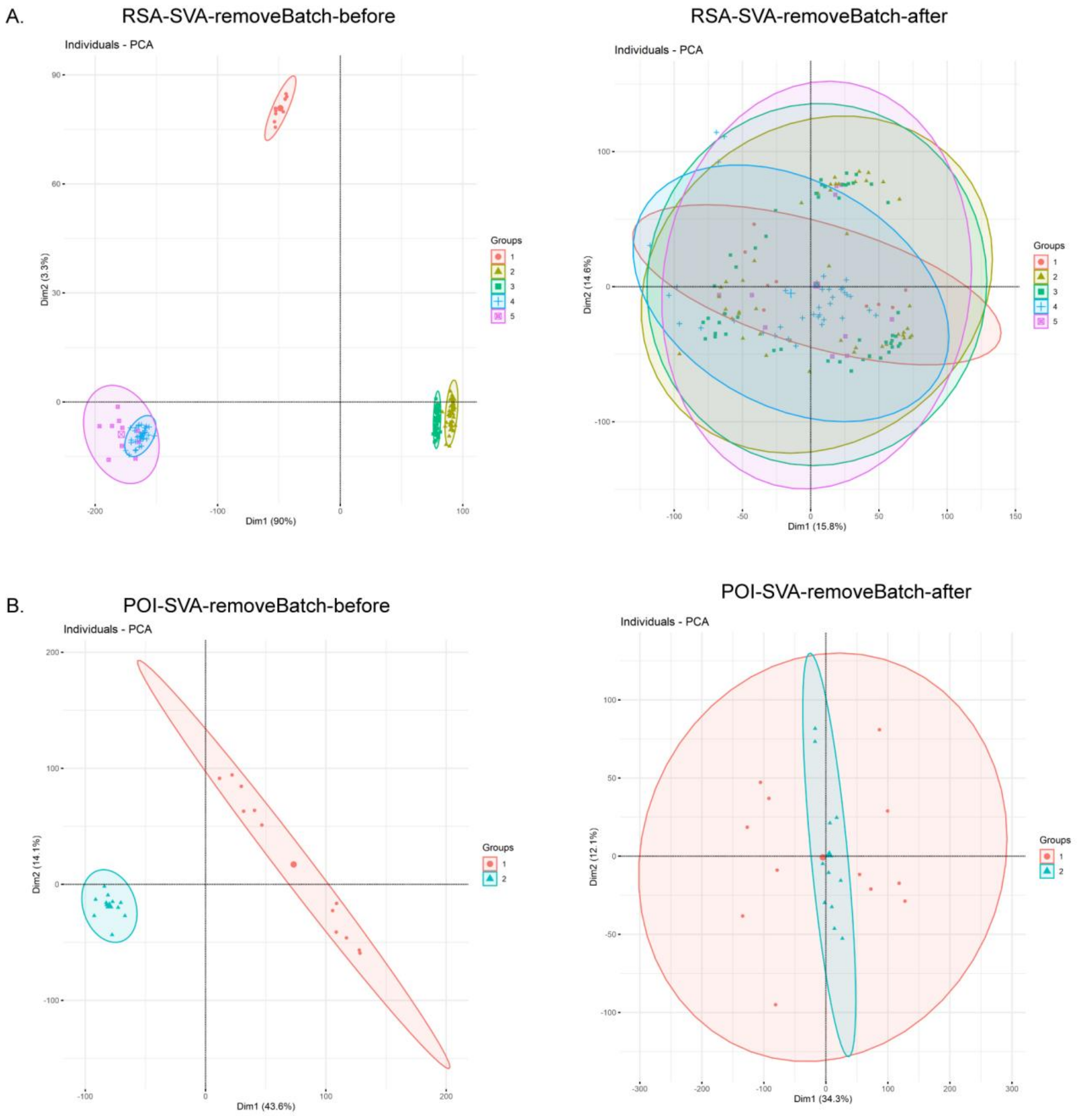

Supply Fig.1 (A) Schematic diagram of PCA before and after eliminating batch effects in RSA dataset **(B)** Schematic diagram of PCA before and after eliminating batch effects in POI dataset

Supplementary Figure 2.

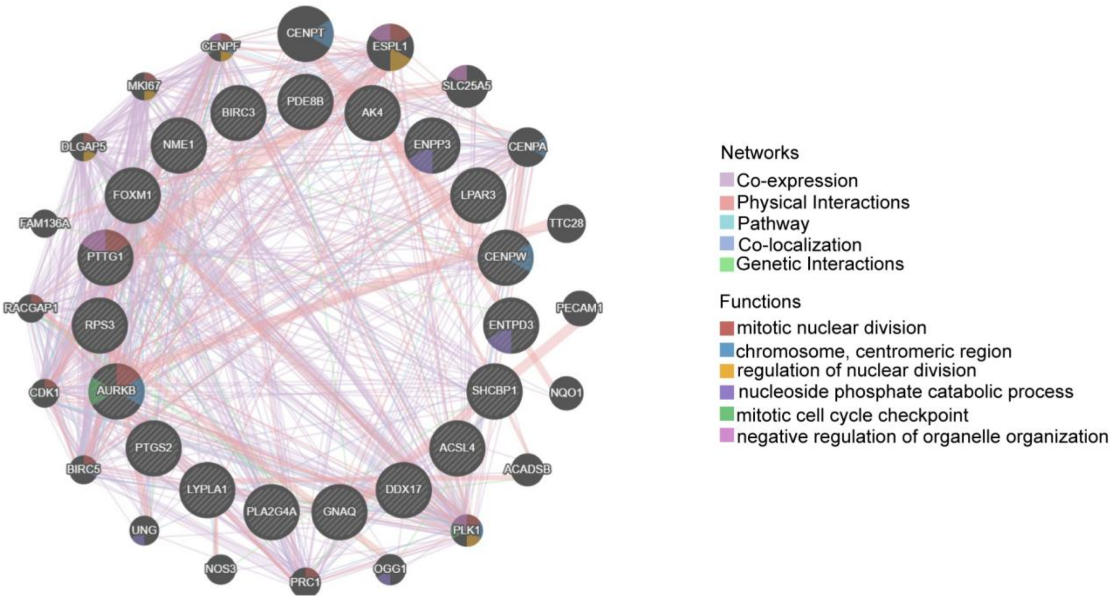

Supply Fig.2 Target gene co－expression network in GeneMANIA database.

Supplementary Figure 3.

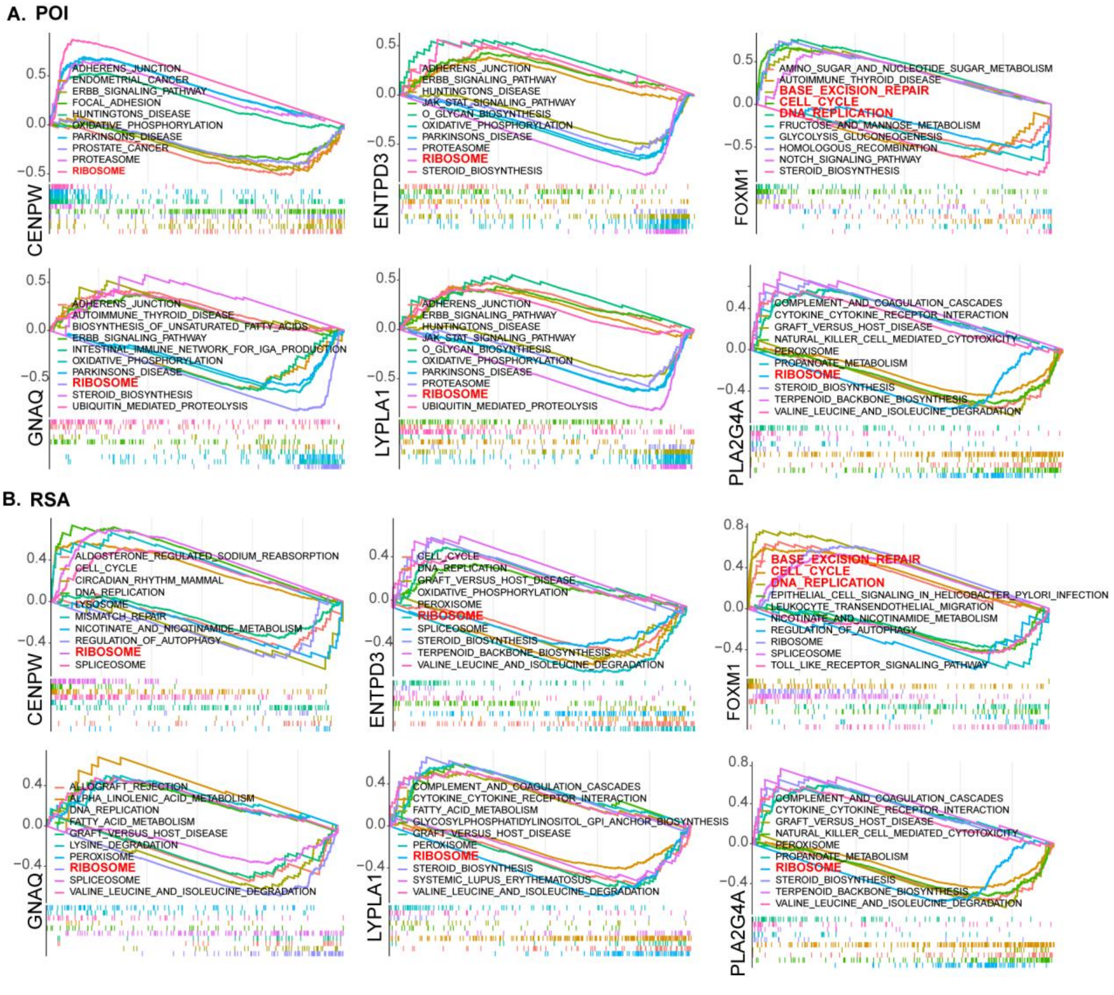

Supply Fig.3 GSEA identifies the signaling pathways that are impacted by the target genes. **(A).**

The main signaling pathways that are significantly enriched by the high expression of the target genes in the POI. **(B).** The main signaling pathways that are significantly enriched by the high expression of the target genes in the RSA.

Supplementary Figure 4.

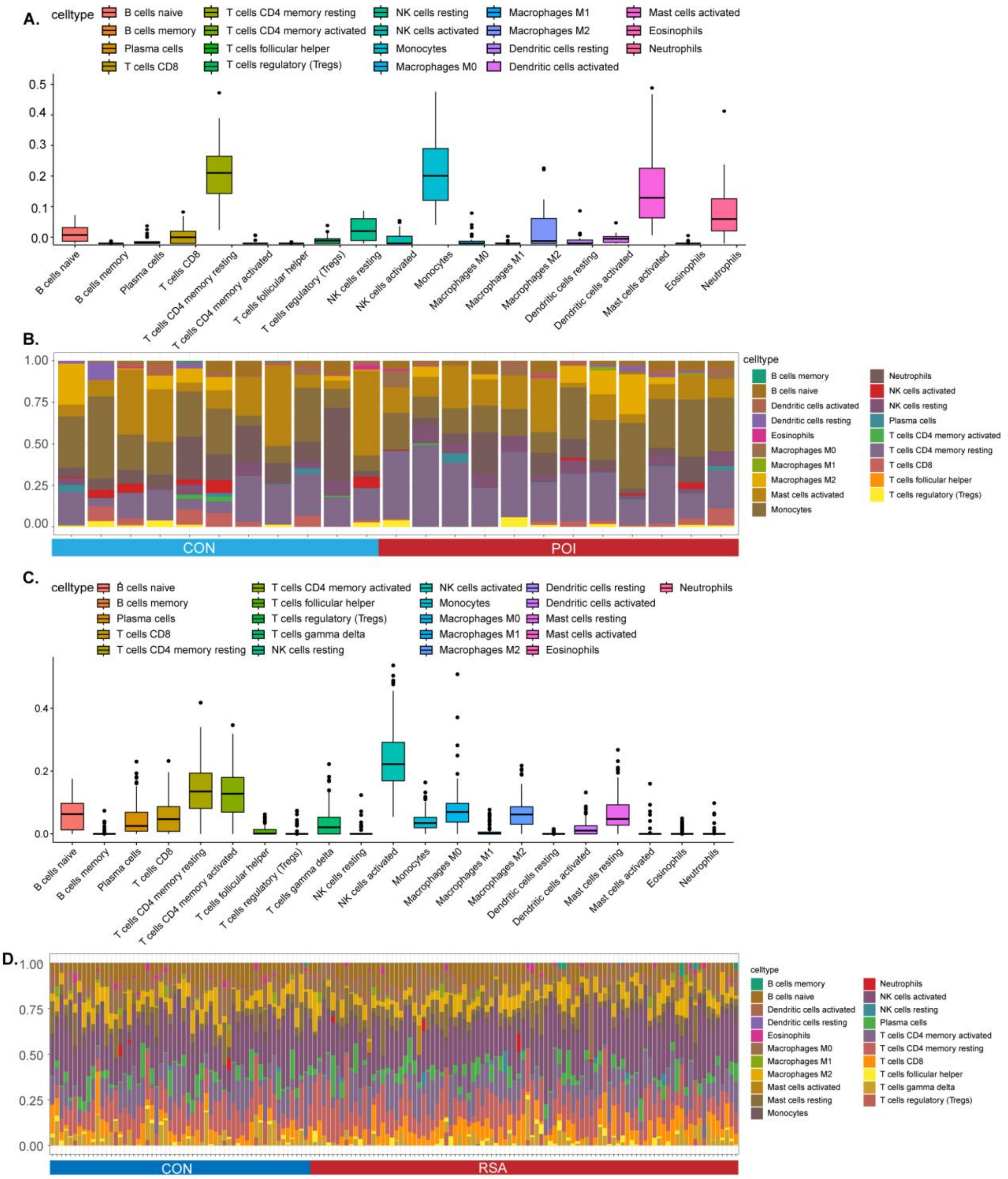

Supplementary Fig.4 POI and RSA immune cell composition. **(A).** Box plot of immune cell distribution in the POI group. **(B).** Infiltrating immune cells were plotted in a stacked bar chart for

the POI group. **(C).** Box plot of immune cell distribution in the RSA group. **(D).** Stacked bar chart showed the RSA group' s characteristics of infiltrating immune cells.

Supplementary Table 1. POI Clinical Sample Statistics

	Control(n=30)	RSA(n=30)	P Value
**Age (year)**	29.1±2.8	30.5±3.2	P=0.220
**BMI (kg/m^2)**	22.9±1.8	23.4±2.1	P=0.290
**Abortion Number**	0.0±0.0	3.5±0.8	P<0.001
**Gravida Number**	2.3±1.0	2.7±0.3	P=0.5130
**Para Number**	2.1±0.2	0.0±0.0	P<0.0001

Supplementary Table 2. RSA Clinical Sample Statistics

	Control(n=15)	POI(n=15)	P Value
**Age (year)**	32.5±3.2	33.0±2.2	P=0.4676
**BMI (kg/m^2)**	20.2±3.0	21.3±2.3	P=0.5236
**FSH (U/L)**	6.3±2.9	31.5±6.7	P<0.0001
**AMH (ng/ml)**	4.7±3.0	0.6±0.3	P<0.0001
**E2 (pmol/L)**	180.0±90.0	75.3±63.6	P<0.0001
**LH (U/L)**	6.5±2.6	21.3±12.1	P<0.0001

Supplementary Table 3. Datasets Detailing

Disease	GSE	Platform	Data Type	Species	Sample Information
RSA	GSE180485	GPL20301	RNA-seq	Human	RSA:36
	GSE183555	GPL21697	RNA-seq	Human	Control:5 VS RSA:5
	GSE165004	GPL16699	RNA-seq	Human	Control:24 VS RSA:24
	GSE111974	GPL17077	microarray	Human	Control:24 VS RSA:24
	GSE26787	GPL570	microarray	Human	Control:5 VS RSA:5
POI	GSE232306	GPL24676	RNA-seq	Human	Control:6 VS POI:6
	GSE201276	GPL20795	RNA-seq	Human	Control:6 VS POI:5

Supplementary Table 4. Primer sequences

Gene	Forward primer	Reverse primer
CENPW	GGCTCCCCGTGGCTTTCTAAAG	AAGCGTTTGTCCTGGACTCTTCTG
ENTPD3	CCCTCTGATCCGTCTGCCCATAG	TCTGGTTGCTGAACACAGGTATGC
FOXM1	GAAGAACTCCATCCGCCACAACC	TGCTGCTGCTTAAACACCTGGTC
GNAQ	TGGACCTGAACCCAGACAGTGAC	CTTGACGGCAGCAAAGACAAAGC
LYPLA1	GGGCTTTCACCAGATTCACAGGAG	GCACCACCGATAGGACCCTGAG
PLA2G4A	ATCACACGAACCCAAAGGCACTG	TACCTTCCCAGCACGTCCTTCTC
GDPDH	GTGGACCTGACCTGCCGTCTAG	GAGTGGGTGTCGCTGTTGAAGTC
